# Effectiveness of aromatherapy for prevention or treatment of disease, medical or preclinical conditions, and injury: protocol for a systematic review and meta-analysis

**DOI:** 10.1186/s13643-022-02015-1

**Published:** 2022-07-26

**Authors:** Sue E. Brennan, Steve McDonald, Melissa Murano, Joanne E. McKenzie

**Affiliations:** School of Public Health and Preventive Medicine, Monash University, 553 St Kilda Road, Melbourne, VIC 3004 Australia

**Keywords:** Aromatherapy, Essential oil therapy, Volatile oils, Natural therapies, Complementary medicine, Complementary and alternative medicine, CAM, Integrative medicine, Massage, Supportive therapy, Systematic review, Meta-analysis

## Abstract

**Background:**

Aromatherapy — the therapeutic use of essential oils from plants (flowers, herbs or trees) to treat ill health and promote physical, emotional and spiritual well-being — is one of the most widely used natural therapies reported by consumers in Western countries. The Australian Government Department of Health (via the National Health and Medical Research Council) has commissioned a suite of independent evidence evaluations to inform the 2019-20 Review of the Australian Government Rebate on Private Health Insurance for Natural Therapies. This protocol is for one of the evaluations: a systematic review that aims to examine the effectiveness of aromatherapy in preventing and/or treating injury, disease, medical conditions or preclinical conditions.

**Methods:**

*Eligibility criteria*: randomised trials comparing (1) aromatherapy (delivered by any mode) to no aromatherapy (inactive controls), (2) aromatherapy (delivered by massage) to massage alone or (3) aromatherapy to ‘gold standard’ treatments. *Populations*: any condition, pre-condition, injury or risk factor (excluding healthy participants without clearly identified risk factors). *Outcomes*: any for which aromatherapy is indicated.

*Searches*: Cochrane Central Register of Controlled Trials (CENTRAL), with a supplementary search of PubMed (covering a 6-month lag period for processing records in CENTRAL and records not indexed in MEDLINE), AMED and Emcare. No date, language or geographic limitations will be applied.

*Data and analysis*: screening by two authors, independently (records indexed by Aromatherapy or Oils volatile or aromatherapy in title; all full text) or one author (remaining records) with second author until 80% agreement. Data extraction and risk of bias assessment (ROB 2.0) will be piloted by three authors, then completed by a single author and checked by a second. Comparisons will be based on broad outcome categories (e.g. pain, emotional functioning, sleep disruption) stratified by population subgroups (e.g. chronic pain conditions, cancer, dementia) as defined in the analytic framework for the review. Meta-analysis or other synthesis methods will be used to combine results across studies. GRADE methods will be used to assess certainty of evidence and summarise findings.

**Discussion:**

Results of the systematic review will provide a comprehensive and up-to-date synthesis of evidence about the effectiveness of aromatherapy.

**Systematic review registration:**

PROSPERO CRD42021268244

**Supplementary Information:**

The online version contains supplementary material available at 10.1186/s13643-022-02015-1.

## Background

In 2015, the Australian Government conducted a *Review of the Australian Government Rebate on Natural Therapies for Private Health Insurance (2015 Review).* Underpinned by systematic reviews of evidence for each natural therapy, one of the findings from the 2015 Review was that there was no clear scientific evidence that aromatherapy was effective. This protocol for a systematic review of aromatherapy describes the methodology for one of a suite of independent evidence evaluations commissioned by the Australian Government Department of Health (the Department) via the National Health and Medical Research Council (NHMRC) to update the evidence and inform the *Review of the Australian Government Rebate on Private Health Insurance for Natural Therapies 2019-20* (2019-20 Review) [1].

Aromatherapy is one of the most widely used natural therapies reported by consumers in Western countries. A systematic review of 89 surveys (97,222 participants), estimating the prevalence of complementary medicine (CM) use by consumers in the UK, found that aromatherapy was the third most popular CM from among 28 different therapies [2]. In Australia, a cross-sectional survey examining consultation with complementary therapists and use of complementary medicine products found that about half of all respondents (1016/2025 adults) used complementary medicines [3, 4]. Aromatherapy oils were used by 11% of respondents (*N* = 224/2019), and 3.9% of respondents had visited an aromatherapist (*N* = 79/2019) [4]. Based on the average spending on complementary medicines reported in this survey, the study authors estimated the total expenditure on aromatherapy oils in Australia to be AUD 250 million in the previous 12 months (2016–2017) [3].

### Description of the intervention

Aromatherapy is the therapeutic use of essential oils from plants (flowers, herbs or trees) to treat ill health and promote physical, emotional and spiritual well-being [1, 5, 6]. The name ‘aromatherapy’ suggests that treatments are delivered directly or indirectly through the olfactory system and that ‘aroma’ is central to therapeutic action. However, there are multiple modes of administration, and these include treatments intended to act through direct contact with the skin and inhalation into the lungs (rather than through an ‘aroma’ inhaled through the olfactory system). The inclusion of such therapies within the scope of aromatherapy practice has led some professional groups to suggest that a more apt description is ‘essential oil therapy’ [7].

#### Active ingredients and choice of essential oils

Although the scope of aromatherapy practice varies, the use of essential oils is central to all definitions [6–10]. Essential oils are volatile oils extracted using steam distillation or mechanical expression from aromatic plants [6, 11]. While it is possible to extract essential oils using solvents (‘absolutes’) and to produce synthetic versions of some oils, aromatherapists generally consider that these are not true essential oils and are therefore unsuitable for therapeutic use [6, 11].

Essential oils vary greatly in their molecular composition. This composition determines the aroma of each oil, and the pathways by which it is absorbed, distributed and metabolised to produce effects [6, 11]. Aromatherapists tailor treatments to individual needs, selecting essential oils, and their mode of application, based on anticipated therapeutic properties for the targeted condition [1, 6].

#### Mode of administration and dose

Inhalation through passive diffusion in the air (e.g. through mist or heat diffusers, steam vaporisation) and direct inhalation (e.g. individual inhalers, steam inhalation) can be used as the primary mode of administering essential oils. Topical application of diluted essential oils to the skin is also common [6]. The intention of topical application may be to produce local effects at the point of administration (e.g. to alleviate pain in a joint) and to mediate effects through inhalation (whether through the lungs or olfactory system) or through skin absorption. Massage is a common co-intervention used with topical application of essential oils. While massage may have a therapeutic effect when used independently of essential oils, it is generally described as an ‘integral’ part of aromatherapy treatment [7]. For topical application, essential oils are diluted in a carrier oil, usually vegetable or nut oil (e.g. sweet almond oil, grapeseed, jojoba oil) [12]. These carrier oils differ from essential oils in that they contain fatty acids, vitamins and minerals, and are believed to aid absorption of the essential oil through the skin [12].

Limiting the dose or concentration of essential oils is considered an important means of avoiding systemic toxicity or adverse effects, such as skin irritation or sensitivity [11, 12]. The typical dose of essential oil used for therapeutic purposes varies depending on indication, and the oil and route of administration, but is generally in the range of a 2.5–5% dilution of essential oils for topical use [11]. Lower concentrations (i.e. higher dilutions) are recommended for some population groups, including women who are pregnant, children and people with conditions or receiving treatments/medications that may put them at greater risk of adverse effects (e.g. people with skin conditions or damage; people undergoing radiotherapy; people with asthma) [7, 11].

Although other routes of administration are sometimes used, professional associations for aromatherapists in Australia, the UK, Canada and the USA have position statements recommending against ingestion of essential oils, internal use (on or near mucous membranes) and the use of undiluted essentials oils on the skin [7–9].

#### Practitioners of aromatherapy and regulation

Aromatherapy is practised by natural therapists, including aromatherapists, naturopaths and massage therapists. It is also an increasingly common professional education option for nurses, allied health professionals and those working in sectors such as palliative care.

Aromatherapy practice is not regulated by the Australian Health Practitioner Regulation National Law, which means there is no requirement for professional registration of practitioners of aromatherapy [13, 14]. The International Aromatherapy and Aromatic Medicine Association (IAAMA) offers membership to aromatherapy practitioners in Australia who have completed accredited training through the National Quality Training Framework [15]. The IAAMA, and other associations for natural therapists in Australia, also set standards for practice and ethical conduct and have requirements for continuing professional education [15, 16]. Some professional associations have safety guidelines and position statements aimed at preventing the use of contraindicated oils, unsafe therapies and treatments that are not widely accepted by the profession (for examples, see [7–10]).

In the 2016–2017 cross-sectional survey examining use of complementary medicine products, only a minority of those who reported therapeutic use of aromatherapy oil consulted a complementary medicine practitioner (12.5%) for a prescription, whereas self-prescription was common (43%) [3]. Indeed, part of the appeal of aromatherapy may be the accessibility of essential oils, which do not require a prescription. The Australian Government provides a safeguard for consumers by regulating essential oils intended for therapeutic use through the Therapeutic Goods Administration (TGA). However, most essential oils are designated as lower risk medicines, which means they are assessed by the TGA for quality and safety, but not effectiveness [17].

### How aromatherapy might work

The research literature and guidance on aromatherapy describes multiple theories of how aromatherapy works (for examples, see [6, 7]). This is perhaps unsurprising given that the exact mechanism by which aromatherapy brings about effects is likely to differ according to the molecular composition of the essential oil and the mode of administration. Similarly, the mechanism of action may vary across outcomes. For example, the mechanism(s) through which aromatherapy might relieve pain may be different from the mechanism for relieving nausea and vomiting [18]. If massage is used as a co-intervention, then the interaction between massage, the essential oil and the carrier oil may also influence the mechanism [6, 12]. Research on these mechanisms comes predominantly from mainstream neurophysiological research on olfaction and pharmacological research. Some is specific to essential oils, but very little originates from literature on aromatherapy [6]. This research is comparatively recent, and evidence about the mechanisms of action for specific oils and modes of delivery is limited [6, 19].

The prevailing description of how aromatherapy works — and one that aligns intuitively with the practice of aromatherapy — is that aromatherapy acts through the olfactory system. Volatile molecules in the aromatherapy oil (the odorant) interact with receptors in the nose, generating an electrical signal to the brain that triggers the perception of smell [6, 19, 20]. This perception includes responses initiated in the limbic system, which is involved in controlling memory and emotion, and through which odours are thought to produce effects on mood, alertness, mental stress, arousal and perceived health [6]. Biochemical or physiological pathways are likely to mediate the effects of essential oils applied to the skin, where either local or systemic effects may be possible depending on whether the active component diffuses through the skin [19]. Some of these effects are suggested to arise from antibacterial, anti-inflammatory and analgesic properties of essential oils [6, 21, 22].

Aromatherapy professional associations also describe a pathway involving an ‘energetic’ or spiritual response. Such mechanisms are described as a ‘vibrational interaction’ between the active component of the essential oil and ‘the energy flows within the body’ [7]. It is unclear whether this pathway relates to the disproven theory that posits a vibrational mechanism of olfaction in which the olfactory system detects molecular vibrations of odour molecules [7, 20, 23].

### Description of conditions for which aromatherapy is used

Although texts on aromatherapy describe a breadth of clinical indications, aromatherapy is often used to treat symptoms of a condition and the side effects of treatment rather than the underlying condition. Examples include the use of aromatherapy to alleviate pain, symptoms of anxiety (that occur as a reaction to stress), low mood, sleep disturbance, behavioural disturbance, vomiting and nausea, and fatigue [6, 24–27]. These indications align with the most commonly treated conditions reported in a 2015 survey completed by 36 practising aromatherapists in Australia [14, 28]. Stress was the condition most frequently reported as ‘often treated’ (by 79% of aromatherapists). Musculoskeletal conditions associated with chronic pain were also frequently reported as often treated, especially neck (64% of aromatherapists), arthritis (54%), sciatica (42%) and knee pain (42%). Other conditions that were reported as ‘often treated’ were headache and migraine (66%), mental health conditions (40%), insomnia (47%), sports injury (27%), cancer (24%) and palliative care (21%).

There is a particular interest in using aromatherapy in circumstances where mainstream interventions may not provide satisfactory relief of symptoms, for example for people with unremitting chronic pain, cancer or advanced disease (not amenable to cure) [6, 25, 29, 30]. Among people with cancer and advanced disease, aromatherapy is used as a form of supportive care to enhance physical and emotional well-being, in addition to alleviating specific symptoms [6, 25, 29, 30]. In other cases, aromatherapy is used as an alternative or adjunctive therapy by those seeking to avoid pharmacological or invasive treatment. For example, aromatherapy has been used to ameliorate behavioural and sleep disturbances among people with dementia [24], to relieve pain during labour [31] and to treat postoperative nausea and vomiting [32]. These treatments may be delivered in a range of healthcare settings (primary, acute and subacute care), with varying levels of integration with conventional providers [33].

Because aromatherapy is often sought or prescribed for relief of symptoms, those receiving aromatherapy for the same indication may have very different underlying conditions (e.g. cancer, arthritis, chronic insomnia) or be undergoing different treatments (e.g. surgery, chemotherapy, minor procedures). Examining the effects of aromatherapy on outcomes for a particular condition may be of interest in some circumstances, but for many commonly treated symptoms or side effects, there is no clear clinical rationale for why the effects of aromatherapy would differ importantly by condition. Where this is the case, a broad synthesis across conditions addresses whether there is a consistent effect for the outcome of interest (benefit, little or no effect, harm), in addition to enabling exploration of whether the effect of aromatherapy differs by condition (e.g. smaller or larger effects).

### Why it is important to do this review

This systematic review will inform the Australian Government’s Natural Therapies Review 2019-20, which is evaluating evidence of the clinical effectiveness of 16 therapies (including aromatherapy). The conclusion from the evidence evaluation conducted on aromatherapy for the *2015 Review* was that ‘there was no clear evidence demonstrating efficacy of aromatherapy’ [34]. The evidence evaluation used overview methods, synthesising results from 20 systematic reviews published up to May 2013. Of the primary studies included in these systematic reviews (*N* = 45), all but one were published prior to 2012. Since the completion of the original evidence evaluation, there has been substantial growth in published research on aromatherapy. A bibliometric analysis of scientific articles on aromatherapy found a steady increase in the number of primary studies and reviews from 1995 to 2014 [35]. Of the 549 research articles published in this period, a third (*N* = 190) were published between 2012 and 2014. This finding marries with claims that there may be evidence available (either published in the last 5 years or not incorporated in systematic reviews at the time the overview was conducted) that may change the conclusions about the effects of aromatherapy [1]. In contrast to the 2015 aromatherapy evidence evaluation, this review will examine evidence from eligible primary studies published from database inception until the date of the last search for this systematic review.

### Objectives

**Fig. 1:**
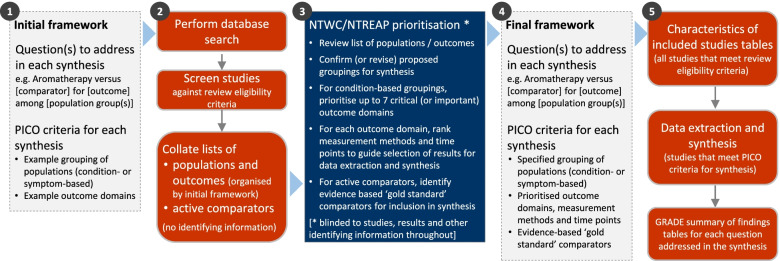
Staged approach for developing the analytic framework for this review

The overall objective of this systematic review is to examine the evidence for the clinical effectiveness of aromatherapy in preventing and/or treating injury, disease, medical conditions or preclinical conditions [1]. The review will focus on outcomes (and underlying conditions) for which aromatherapy is commonly sought or prescribed in Australia, and which are relevant to the 2019-20 Review of the Private Health Insurance rebate. The specific objectives of the review follow (framed as questions). Examples of potentially relevant outcome domains and conditions are included to illustrate the breadth of questions to be addressed in the synthesis. These questions will be refined through a staged prioritisation process (‘Methods’ section, Fig. 1) to align with priorities for the 2019-20 Review, ensure a consistent approach across the evidence evaluations of natural therapies (where appropriate) and make best use of available evidence.

#### Primary objectives

1. What is the effect of *aromatherapy* compared to *no aromatherapy (inactive controls)* (see the section ‘Types of interventions’ — Comparisons) among people with any condition, pre-condition, injury or risk factor on outcomes for which aromatherapy is indicated? (for example, acute pain, emotional functioning and well-being, sleep disruption, behavioural disturbances, health-related quality of life)
2. What is the effect of *aromatherapy plus massage* compared to *massage alone* among people with any condition, pre-condition, injury or risk factor on outcomes for which aromatherapy is indicated? (examples as per objective 1)

#### Secondary objectives

3. What are the effects of *aromatherapy* for each underlying condition, pre-condition, injury or risk factor? (for example, effects on sleep disruption among people undergoing palliative care, people with chronic insomnia, people with chronic pain or people with dementia)
4. What are the effects of *aromatherapy* compared to *evidence-based ‘gold standard’ treatments*? (see the section ‘Types of interventions’ — Comparisons)
5. What evidence exists examining the effects of aromatherapy compared to other active comparators? (i.e. not massage or a ‘gold standard’)

## Methods

Methods reported in this protocol are based on the *Cochrane Handbook for Systematic Reviews of Interventions* [36]. The GRADE approach will be used to summarise and assess the certainty of evidence arising from this review (see the ‘Data collection and analysis’ section for details). GRADE methods are widely used in systematic reviews and guideline development to ensure a systematic, transparent and common approach to interpreting results [37]. The protocol is reported in accordance with the Preferred Reporting Items for Systematic review and Meta-Analyses Protocols (PRISMA-P) statement [38, 39] with consideration given to the extensively updated guidance for reporting methods for systematic review in the Preferred Reporting Items for Systematic review and Meta-Analyses (PRISMA) 2020 statement [40, 41]. The review has been prospectively registered on the International prospective register of systematic reviews (PROSPERO CRD42021268244).

The methods for this review are designed to accommodate the breadth of evidence about the effects of aromatherapy relevant to the 2019-20 Review and ensure a consistent approach with the other evidence evaluations of natural therapies (where appropriate). To achieve this, we will follow the staged approach summarised in Fig. 1 and elaborated in subsequent sections. We begin with an initial analytic framework (step 1) that will be refined through a prioritisation process. To facilitate this process, we will screen studies against the review eligibility criteria and compile an aggregate list of populations and outcomes, derived from the included studies and organised by the initial framework (step 2). No identifying information will be included (i.e. no study-level information, results, references, number of studies etc.). The NHMRC’s Natural Therapies Working Committee (NTWC) and the Department’s Natural Therapies Review Expert Advisory Panel (NTREAP) will review the list in order to prioritise outcomes and advise on the final framework for the synthesis (step 3), which will be finalised (step 4) prior to proceeding with the review (step 5).

**Fig. 2:**
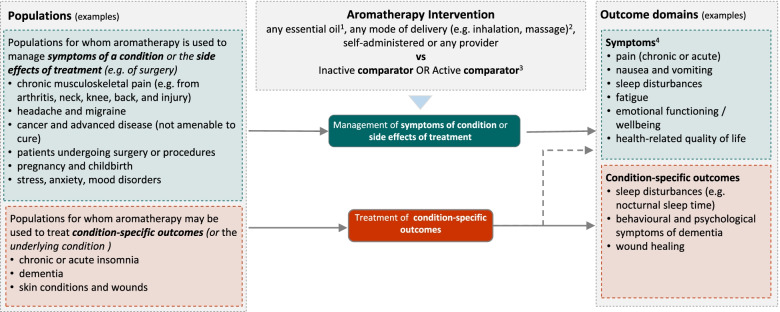
Initial analytic framework for the review. ^1^Excluding oils considered unsafe for therapeutic use in humans. ^2^Excluding ingestion, internal administration or undiluted application to the skin. ^3^Synthesis limited to inactive, massage (when aromatherapy is delivered via massage) and evidence-based ‘gold standard’ treatments. ^4^Symptoms relevant to each population group will vary

Figure 2 shows the initial analytic framework for the review. Example populations and outcome domains are included to convey the breadth of the review, and illustrate possible population and outcome groups for synthesis. These are indicative and not intended to be exhaustive. The framework was informed by research on the outcomes (and underlying conditions) for which aromatherapy is commonly sought or prescribed in Australia, a scoping search of studies evaluating aromatherapy, the wider literature on aromatherapy and consideration of frameworks for classifying disease and outcomes [42, 43]. Details for each population, intervention, comparator, outcomes (PICO) element follow (see the ‘Criteria for considering studies for this review’ section).

### Criteria for considering studies for this review

#### Types of studies

Randomised controlled trials (RCTs) are eligible for inclusion in the review (including individually and cluster randomised and cross-over trials).

Controlled trials in which the allocation sequence did not include a truly random element, was predictable or was not adequately concealed from investigators are eligible as long as there was an attempt to have some kind of ‘randomisation’ to groups. Examples include studies using methods for sequence generation based on alternation, dates (of birth or admission) and patient record numbers [44].

##### Exclusions

- Non-randomised studies of interventions (NRSIs)
- Studies described as ‘randomised trials’ or ‘controlled clinical trials’, but in which decisions about the allocation of participants to treatment groups were (1) made by clinicians or participants or (2) based on the availability of the intervention. These studies lack any ‘attempt’ at randomisation and, as such, are likely to be at high risk of selection bias whereby participants may be selected into groups based on factors that are prognostic of outcomes (which may introduce confounding). These studies will be treated as non-randomised studies.
- Studies for which available reports have not been peer reviewed (grey literature)

The decision to exclude non-randomised studies was informed by scanning results from a scoping search of the Cochrane Central Register of Controlled Trials (CENTRAL) (see the ‘Electronic searches’ section) and results of a more limited search of PubMed using a resource on the National Institute of Health National Centre for Complementary and Integrative Health website [45]. The scoping search of CENTRAL retrieved in excess of 500 potentially eligible trials, from which we anticipate a high proportion (100–200) will meet eligibility criteria for the review. Given the likely size and breadth of the evidence base, and the proposed structure for the synthesis, any effect of aromatherapy on health outcomes should be detectable from randomised trials. The inclusion of NRSIs is unlikely to increase certainty of the results from a body of randomised trial evidence of this size or alter the conclusions of the review.

##### Date and language restrictions

There are no restrictions on publication date.

Potentially eligible studies published in languages other than English will not be included in the review, but will be listed according to whether they are likely to be eligible or whether eligibility cannot be determined (see the ‘Selection of studies’ section). The impact of excluding these studies will be considered in the assessment of bias due to missing results (see the ‘Assessment of biases due to missing results’ and ‘Summary of findings tables and assessment of the certainty of the body of evidence’ sections).

#### Types of participants

Studies involving participants with any disease, medical condition, injury or preclinical condition are eligible for the review. This includes healthy participants with clearly identified risk factors (e.g. biomedical, health behaviours or other). There are no restrictions on age.

We expect that studies will include participants that fall within broad population groups, such as those shown in Fig. 2. These are indicative groups, included to illustrate the breadth of populations eligible for the review and possible groupings for synthesis. Decisions about which groups to include in the final analytic framework will be made through the prioritisation process (Fig. 1). This process may lead to changes and additions to the population groups (i.e. broader, narrower or new groups).

##### Basis for grouping

Because of the broad range of indications for aromatherapy (e.g. management of symptoms of a condition or side effects of treatment for a condition, versus treatment of an underlying condition), the basis of the population groups shown in the initial analytic framework varies. Some are grouped by symptom (e.g. chronic pain), some by treatment for an underlying condition (e.g. surgery and other procedures) and others by the underlying condition (e.g. chronic insomnia, dementia). The groupings are based on International Classification of Diseases 11th Revision (ICD-11) codes and encompass conditions identified in aromatherapy literature and the Practitioner Research and Collaboration Initiative (PRACI) survey as often treated by aromatherapists [14, 28].

Participants who are otherwise healthy will be considered to have a clearly identified risk factor if participating in a trial aimed at prevention of a disease/condition for which their risk factor is an eligibility criterion (e.g. individuals with signs or symptoms of work-related anxiety who are confirmed to be at risk of developing a clinically diagnosed anxiety or fear related disorder).

##### Exclusions

- Healthy populations seeking health improvement

Studies that include both healthy participants and participants eligible for the review will be included if separate data are available or a majority of participants meet the review eligibility criteria as per guidance in the *Cochrane handbook* [46]. For the latter, we will consider implications for the applicability of study findings in the Grading of Recommendations, Assessment, Development and Evaluation (GRADE) assessment.

While studies involving any population will be included in the review (except for the specific exclusions above), if the number of eligible studies for synthesis is unmanageable, the synthesis may be limited to populations (conditions) most relevant to aromatherapy practice in Australia. Such decisions will be made through the prioritisation process (Fig. 1), guided by data about practice in the Australian context (e.g. practitioner or patient surveys that report reasons for use in Australia). Studies excluded from the synthesis will be included in an evidence inventory (objective 5).

#### Types of interventions

For the purpose of this review, aromatherapy is defined as ‘Administration of aromatherapy oils by inhalation, diluted topical use and massage’ [1].

Except for the specific exclusions below, aromatherapy treatments will be eligible irrespective of the type of essential oil, carrier or dispersant, mode of delivery or route of administration, whether self-administered or provided by a practitioner, the training or qualifications of the practitioner and the dose and duration of treatment. More details about each of these intervention features are considered under the ‘Data extraction and management’ section. See also Appendix 3, Additional file 1.

##### Excluded therapies

In line with the recommendations from aromatherapy professional associations in Australia and internationally [7–10], we will exclude interventions in which an essential oil is:

- Ingested or administered internally (e.g. oral, vaginal, rectal or other internal routes of administration)
- Applied undiluted to the skin
- Considered unsafe for therapeutic use in humans

##### Comparisons

1. Aromatherapy (delivered by any mode, including massage) versus any inactive comparator (placebo/sham, no intervention, wait list control, usual care)
2. Aromatherapy delivered by massage versus massage alone (this comparison is included to isolate the effects of aromatherapy)
3. Aromatherapy (delivered by any mode) versus evidence-based gold standard treatment(s) (see below for selection method)
4. Aromatherapy (delivered by any mode) versus other active comparators (for inclusion in evidence inventory only, not the synthesis — see below)

These comparisons will form the basis of separate syntheses (meta-analyses), each considering an outcome domain with studies grouped within by population group (where appropriate; see Fig. 2 for examples). Where a study includes multiple arms, with at least one eligible comparator (e.g. a placebo control arm), we will include the eligible comparison(s).

For comparison 3, evidence-based gold standard treatments will be identified through the prioritisation process (Fig. 1, step 3). We will provide the NTWC and NTREAP with a list of active comparators identified from included studies (Fig. 1, step 2). Studies with active comparators will not contribute to the synthesis except in the exceptional circumstance where the NTWC considers that the comparator intervention is an accepted, evidence-based ‘gold standard’ of care for the population in the studies, and there are studies suitable for conducting a synthesis (meta-analysis) (i.e. comparable PICO criteria, low risk of bias). These judgements will be made blinded to the studies and study results, to the fullest extent possible. For studies involving other active comparators, we will provide an inventory of available evidence, tabulating a brief description of the characteristics of PICO for each study.

##### Exclusions

In line with the main review objective, which is to examine the effects of aromatherapy rather than the comparative effects of different aromatherapy treatments, we will exclude head-to-head comparisons of aromatherapy from the review (see exceptions below). For example, we will exclude studies where the only comparator is:

- Another essential oil or preparation of an essential oil (e.g. lavender versus ginger)
- A different dilution or dose of the same essential oil
- A different carrier of the same essential oil
- A different mode of delivery of the same essential oil (e.g. two different modes of inhalation; inhalation versus massage)
- Where the person administering the therapy has a different qualification, specialisation or skill level (e.g. aromatherapists versus other health professional; this includes comparisons of self-administration versus administration by a practitioner)
- Or combinations of the above

#### Types of outcomes

Outcomes eligible for this review are those that align with the reasons why aromatherapy is sought by patients and prescribed by practitioners. In principle, this may include any patient-important outcome that helps elucidate the effects of aromatherapy on an underlying condition or its symptoms, recovery, rehabilitation or prevention of disease among people with specific risk factors or pre-conditions.

Example outcome domains are shown in Fig. 2. Appendix 2 in Additional file 1 provides examples of specific outcomes within each domain, and populations to which the outcome domain may be relevant. Because aromatherapy is often used for the management of symptoms of a condition or side effects of treatments (anxiety, pain, nausea and vomiting), ‘symptoms’ are separated from ‘condition-based’ outcomes (the latter encompassing outcomes of relevance when aromatherapy is used to treat the underlying condition). The example outcome domains are intended to illustrate the breadth of outcomes likely to be important for understanding the effects of aromatherapy across a wide range of conditions, as identified from the PRACI survey of the conditions often treated by aromatherapists in Australia [14, 28] and the wider literature on aromatherapy.

The initial grouping of broadly related outcomes within each domain (Fig. 2) is based on ICD-11 codes and the Core Outcome Measures in Effectiveness Trials (COMET) outcome taxonomy [42, 43]. These systems provide a widely agreed and understood structure for categorising different outcomes and address the fact that there is not always a clear distinction between outcomes and conditions. For example, some outcome domains closely match the primary diagnosis for particular patients (e.g. insomnia, chronic pain, anxiety), but are a symptom or side effect of treatment for other conditions (e.g. cancer or surgery).

#### Prioritisation and selection of outcomes for summary and synthesis

##### Outcome prioritisation

To accommodate the breadth of relevant outcomes, the outcome domains and population-specific outcomes for inclusion in the synthesis will be determined through the prioritisation process (Fig. 1).

To prioritise the most important outcomes for this review:

- We will compile a list of specific outcomes from included studies and example outcome measures (without results or identification of studies).
- Outcomes in the list will be categorised by the outcome domains and population groups in Fig. 2. Outcomes that fall outside the proposed outcome domains will also be listed.
- The NTWC will be asked to indicate whether each of the listed outcome domains (or specific outcomes) is critical, important or of limited importance for understanding the effects of aromatherapy on each population group. Only critical and important outcomes will be considered in the review.

##### Outcome selection

From each study, we will select only one outcome per outcome domain for data extraction (results), risk of bias assessment and inclusion in the summary and synthesis.

- An initial hierarchy of population-specific outcomes and measures will be presented to the NTWC for discussion and approval (e.g. a hierarchy of pain outcomes and measures for osteoarthritis).
- Where possible, the initial hierarchy will be based on outcome hierarchies used in published Cochrane reviews, systematic reviews of measures that provide evidence of the relevance and validity of measures, and core outcome sets.
- We will also seek advice on the most relevant time point for outcome measurement. This is likely to be immediately post-intervention (end of the intervention period if multiple treatments). In some instances, the longest follow-up may be relevant (e.g. chronic pain).
- The agreed hierarchy of population-specific outcomes measures and time points will be used to select the most relevant and valid measure of each outcome domain available from each study for inclusion in the synthesis.

Exclusions:

- Experience of care (e.g. satisfaction)
- Safety
- Quality
- Economic outcomes

Studies will not be excluded from the synthesis/reporting of results based on outcome, except where it is possible to confirm that the study did not measure an outcome eligible for the review (e.g. from a registry record or protocol).

### Search methods for identification of studies

#### Electronic searches

The primary source of studies will be the Cochrane Central Register of Controlled Trials (CENTRAL), the most comprehensive source of published and unpublished reports of randomised trials. Most CENTRAL records are derived from regular searches of bibliographic databases, such as MEDLINE, Embase and the Cumulative Index of Nursing and Allied Health Literature (CINAHL). Records from clinical trial registers (ClinicalTrials.gov and WHO International Clinical Trials Registry Platform [ICTRP]) and the specialised registers maintained by Cochrane groups also make up a substantial proportion of records in CENTRAL.

As part of Cochrane’s centralised search service, the major bibliographic databases and trial registers are searched monthly and, using a combination of automation and crowd screening, records deemed to be reports of randomised trials are added to CENTRAL [47]. In a recent evaluation, over 97% of studies included in Cochrane reviews were retrieved by Cochrane’s centralised search service [48]. Given the large volume of studies we anticipate will be eligible, we are confident that limiting our search to CENTRAL, with supplementary searches of PubMed and the Allied and Complementary Medicine Database (AMED), will capture a very high proportion of all relevant studies.

The proposed search strategy for CENTRAL includes the key thesaurus terms and text words for aromatherapy, as well as more peripheral terms, such as essential oils (see Appendix 1 in Additional file 1). The most commonly used essential oils are included as text words in their own right. This list of oils was compiled from (1) studies included in the overview of aromatherapy for the 2015 Review [34] and (2) the broader aromatherapy literature [6, 21, 22, 24–27, 31]. To ensure no commonly used essential oils were missing from the list, we examined a sample of 272 abstracts from a PubMed Clinical Query for aromatherapy (category: ‘Therapy’, scope: ‘Narrow’). We will not limit the search by language, year of publication or publication status.

Since there is a lag between when records are processed by Cochrane and when they appear in CENTRAL, we will run a search in PubMed for records added in the previous 6 months. In addition, to ensure we include records available in PubMed but which are not indexed in MEDLINE, we will search PubMed for all years, limited to the non-MEDLINE subset (see Appendix 1 in Additional file 1).

We will also search AMED and Emcare via Ovid as these databases are not ones that Cochrane searches centrally.

Scoping searches reveal that about 500 records in CENTRAL (excluding records from ClinicalTrials.gov or WHO ICTRP) are either indexed with the Medical Subject Headings (MeSH) or Emtree term Aromatherapy or have aromatherapy as a text word in the title. A further 1300 records are retrieved with the remaining search terms.

#### Searching other resources

We will screen studies provided by the public and key stakeholders (via the Department), NTREAP and NTWC for eligibility. Where these groups recommend particular systematic reviews, we will examine references for included studies to identify potentially eligible randomised trials.

We will ensure that all randomised trials included in the 2015 evidence evaluation for aromatherapy are considered for inclusion.

We will not examine the reference lists of included studies to identify additional trials (i.e. backward citation searching), nor will we conduct forwards citation searching (i.e. looking for studies that have cited included studies). Empirical studies assessing the value of reference checking (backward citation searching) as part of the systematic review process indicate that it is most useful for areas that are difficult to search electronically (new technologies, cross-disciplinary topics, complex interventions) or for which review authors aim to locate grey literature [49]. Forward citation searching is much less common in systematic reviews [50] and of questionable value [51]. Conducting forward citation searching for the large volume of aromatherapy studies we anticipate including in this review could generate thousands of additional records to screen, with little evidence that we would identify unique studies. This has significant time and cost implications [52]. We anticipate that our search is sufficiently sensitive that we are unlikely to miss important studies, and given the anticipated volume of eligible studies and breadth of the review question, it is unlikely that any missing studies would alter the findings of the review.

### Data collection and analysis

#### Selection of studies

Records from CENTRAL, PubMed and AMED will be imported into EndNote and duplicates removed. All remaining records will be imported into Covidence [53] for screening. Records submitted through the Department, NTREAP or NTWC will be screened to confirm that the type of study is eligible, then non-duplicate records will be imported into Covidence for screening alongside other studies.

We will pilot guidance for title and abstract screening on a sample of 50 records to ensure the eligibility criteria are being applied consistently by three reviewers (SB, MM, SM). If needed, we will amend the screening guidance (but not the eligibility criteria) to enhance consistency. We propose to split title and abstract screening into two phases. Phase 1 records (indexed with the thesaurus terms Aromatherapy or Oils volatile or with aromatherapy in the title) will be screened independently by at least two reviewers. Phase 2 (remaining records) will be screened by one reviewer, with a 10% random sample screened by a second reviewer (with further sampling if needed until 80% agreement is achieved). All records selected for full-text screening will be reviewed independently by two reviewers. Disagreements at either stage of screening will be resolved by consensus among members of the review team. Where disagreement cannot be resolved, advice will be sought from the NTWC (which will be provided with PICO characteristics for the de-identified study).

Studies confirmed as meeting the eligibility criteria, but for which results are not available in a published report, will be included in a list of ‘ongoing studies’.

The following will be included in a list of ‘studies awaiting classification’.

**Fig. 3:**
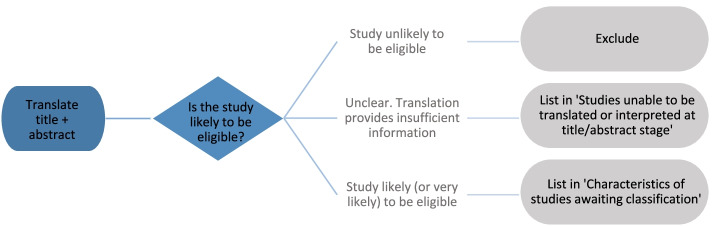
Flowchart showing handling of studies in languages other than English (reproduced from NHMRC framework for natural therapies systematic reviews [54])

- Studies that are only published as abstracts or for which a full report is not available (i.e. we will not seek further information from study authors to confirm eligibility)
- Studies identified by, or submitted to, the review team after the date of the last search
- Studies confirmed as likely to be eligible, but for which no English language translation of the full-text publication is available. Studies for which eligibility cannot be confirmed following translation of the title and abstract using Google Translate will be listed separately (Fig. 3).

Studies that do not meet the eligibility criteria will be excluded and the reason for exclusion will be recorded at full-text screening. These studies will be included in a ‘Characteristics of excluded studies’ table in which the reason for exclusion is reported.

The search and study selection steps will be summarised in a PRISMA flow diagram.

For studies that originated from the call for evidence, NTREAP or NTWC, we will record and report exclusion decisions irrespective of whether the study was excluded during title and abstract screening or full-text review. We will document the flow of these studies through the review in the PRISMA flow chart and annotate tables with the source.

##### Dealing with duplicate and companion publications

Multiple publications to the same study (e.g. protocols, trial registry entries, trial reports) will be identified and linked at the data extraction stage in Covidence systematic review management software [53]. Each study will be given a unique identifier and all linked records cited in the final report. Records will be matched using trial registry numbers. Where these are not available, we will consider author names, trial name, trial location(s) and number of participants.

#### Data extraction and management

Study data will be collected and managed using REDCap electronic data capture tools hosted at Monash University [55, 56]. Three authors (MM, SB or SM) will pre-test the data extraction and coding form on 3–5 studies (as needed to achieve consistent coding), purposefully selected from the included studies to cover the diversity of data types anticipated in the review. One author (SB) will review the extracted and coded data for completeness, accuracy and consistency. Where needed, advice will be sought from the clinical advisor (SG) and biostatistician (JM) to ensure data are extracted as planned. Revisions to the data extraction form and guidance will be made as required to maximise the quality and consistency of data collection.

For each included study, one review author (MM, SB or SM) will extract study characteristics and quantitative data using a pre-tested data extraction and coding form, with a 10% random sample extracted by a second author (with further sampling if needed until 80% agreement is achieved). For studies extracted by a single author, a second author (MM, SB or SM) will independently verify the quantitative data. Discrepancies will be resolved through discussion, and advice sought from the clinical advisor (SG) or biostatistician (JM) if agreement cannot be reached or for more complex scenarios.

We will extract information relating to the characteristics of included studies and results as follows.

1. Study identifiers and characteristics of the study design
  Study references (multiple publications arising from the same study will be matched to an index reference; code as index paper, protocol, registry entry, results paper 1, 2, …)
  Study name, location, commencement date and trial registration number
  Study design (categorised as ‘individually randomised’, ‘cluster randomised’, ‘cross-over’ or ‘other’)
  Funding sources and funder involvement in study
  Financial and non-financial interests declared by investigators
2. Characteristics of each intervention group (including comparator groups)
  Characteristics of the intervention structured by domains of the Template for Intervention Description and Replication (TIDieR) checklist [57] (see Appendix 3 in Additional file 1 for TIDieR domains, codes and an example of coding for aromatherapy)
  Number of participants: randomised to each group, at follow-up for selected outcome, and included in analysis and reasons for loss to follow-up
3. Characteristics of participants
  Participant eligibility criteria (verbatim)
  Age (e.g. mean, median, range)
  Sex
  Population group: coded using categories specified in the final analytic framework for the review (e.g. chronic pain, headache and migraine, cancer and advanced disease (not amenable to cure), surgery or procedures, pregnancy and childbirth, chronic or insomnia, dementia, stress, anxiety and mood disorders)
  Condition: specific underlying condition as described in study (e.g. haematological tumours; rheumatoid arthritis), including information about severity (if relevant)
  Treatment/procedure: applies to studies in which aromatherapy is administered for the relief of symptoms or side effects of a treatment or procedure for an underlying condition (e.g. radiotherapy; bone marrow biopsy). May include pharmacological treatment, surgical, diagnostic or other procedures (as described in study, and coded using categories specified for the review e.g. pharmacological, surgery, minor or major non-surgical procedure)
  Other characteristics of importance within the context of each study
4. Outcomes assessed and results
  Outcomes measured (a list of all outcomes [noting primary outcome(s) for study], categorised according to the broad domains specified in the final analytic framework for the review, or as ‘other’ if none of the outcome domains applies)
  For outcomes selected for inclusion in the summary and synthesis of results:
  Outcome domain: categorised according to the broad domains specified in the final analytic framework for the review (e.g. pain, sleep disturbances, nausea and vomiting, emotional functioning/well-being, behavioural disturbances, cognitive functioning, fatigue, health-related quality of life)
    ° Outcome as described in the included study (verbatim or precis)
    ° Measurement method (e.g. Rotterdam Symptom Checklist, used to measure psychological and physical aspects of quality of life for people with cancer), information required to interpret the measure (scale range and direction, minimally important difference) and time point (exact, and time-frame categorised as ‘immediate’ or ‘longest follow-up’)
    ° Results including summary statistics by group (means and standard deviations, or number of events for cognitive outcomes that have been dichotomised, and sample size), estimates of intervention effect (e.g. mean differences (or adjusted mean differences), confidence intervals, *t*-values, *p*-values or risk ratios/odds ratios for binary outcomes)
    ° Data required to support risk of bias judgements (see the ‘Assessment of risk of bias of included studies’ section) [58]

#### Assessment of risk of bias of included studies

##### Assessment of risk of bias in RCTs

We will assess the risk of bias in included studies using the revised Cochrane ‘Risk of Bias’ tool (RoB 2) for randomised trials [44, 58] for each critical (or important) outcome included in the synthesis. Our assessment will be based on the *effect of assignment to the intervention*.

RoB 2 addresses five domains:

- Bias arising from the randomisation process
- Bias due to deviations from intended interventions
- Bias due to missing outcome data
- Bias in measurement of the outcome
- Bias in selection of the reported result

To promote concordance, the assessment will be piloted by three review authors (MM, SB, SMc) on 3–5 studies until consistent judgements are achieved across a range of scenarios. One review author (MM, SB or SMc) will then apply the tool to the selected results from each study following the RoB 2 guidance [44], and a second author will verify the assessments (SB or SMc). Supporting information and justifications for judgements for each domain (low, some concerns, high risk of bias) will be recorded. We will derive an overall summary of the risk of bias from each assessment, following the algorithm in the RoB 2 guidance [45]. Disagreement between review authors will be resolved through discussion, and a third review author (SB, SM or JM) will adjudicate where agreement cannot be reached. For cluster trials and cross-over trials, we will use the variant of the RoB 2 tool specific for the design [59].

When multiple effects of the intervention using different approaches are presented in the trial report, we will select one effect for inclusion in the meta-analysis and for risk of bias assessment. The selected effect will be chosen according to the following hierarchy, which orders the approaches from (likely) least to most biased for estimating the *effect of assignment to the intervention*: (1) the effect that corresponds to a full intention-to-treat analysis, where missing data have been multiply imputed, or a model-based approach has been used (e.g. likelihood-based analysis, inverse-probability weighting); (2) the effect corresponding to an analysis that adheres to intention-to-treat principles except that the missing outcome data are excluded; (3) the effect that corresponds to a full intention-to-treat analysis, where missing data have been imputed using methods that treat the imputed data as if they were observed (e.g. last observation carried forward, mean imputation, regression imputation, stochastic imputation); or (4) the effect that corresponds to an ‘as-treated’ or ‘per-protocol’ analysis, or an analysis from which eligible trial participants were excluded [58, 59].

#### Measures of treatment effect

We anticipate that many of the outcomes will be continuous (e.g. pain, anxiety) and that varying measurement instruments will be used to measure the same underlying construct across the studies. For this reason, we will quantify the effects of aromatherapy using the standardised mean difference (SMD) (implementing the Hedges’ adjusted *g* version). In trials where a continuous measure has been dichotomised (e.g. a continuous pain scale is dichotomised into improvement or no improvement) and analysed as binary outcomes, we will re-express reported, or calculated, odds ratios as SMDs [60]. For dichotomous outcomes, we will quantify the effects of aromatherapy using risk ratios (RR). Given the wide range of conditions and outcomes in this review, it is not possible to specify specific thresholds for interpreting the size of the effect for each outcome. Given this, we plan to use Cohen’s guiding rules for SMDs where 0.2 represents a small effect, 0.5 a moderate effect and 0.8 a large effect [61]. Where a valid and reliable minimal important difference (MID) is available for a familiar measure of relevance to the population groups in the meta-analysis, we will re-express the SMD in units of the measure and interpret the effect in relation to the MID if feasible to do so [61]. For dichotomous outcomes, we will seek advice from the NTWC on interpreting the size of the effect (seeking agreement on a threshold for a small but important difference).

#### Unit of analysis issues

In this review, unit of analysis issues may arise from non-standard designs (cluster trials, cross-over trials) or from trials with more than two eligible intervention groups. In the following, we outline the methods for making adjustments when necessary. Any adjustments will be documented (e.g. assumed intra-cluster correlation and average cluster size). We will also report when necessary adjustments were unable to be made due to missing information.

For cluster randomised trials that have not appropriately accounted for correlation in observations within clusters, we will attempt a re-analysis. We will do this by inflating the variance of the intervention estimates by a design effect (DEFF). The DEFF is calculated from two quantities — an intra-cluster correlation (ICC) and the average cluster size. Estimates of ICC will be imputed from other cluster trials included in the review, where possible, or by using external estimates from empirical research (e.g. Bell [62]). The average cluster size will be calculated from reported information in the trial.

For cross-over trials where an appropriate paired analysis is not available, we will attempt to approximate a paired analysis by imputing missing statistics (e.g. correlation). Estimates of the missing statistics will be imputed from other cross-over trials included in the review, where possible, or by using external estimates from empirical research (e.g. Balk [63]).

For trials where more than one comparison from the same trial is eligible for inclusion in the same meta-analysis (e.g. lavender oil, ginger oil, control), we will combine intervention groups, where it makes sense to do so; otherwise, we will appropriately reduce the sample size so that the same participants do not contribute more than once.

#### Dealing with missing data

We will not contact trial authors to obtain missing information (e.g. study characteristics, description of conduct of the trial) or aggregate level statistics (e.g. missing standard deviations). However, we will attempt to calculate statistics necessary for meta-analysis using algebraic manipulation of reported statistics (e.g. computing the standard error for the treatment effect from a reported *p*-value). When standard deviations cannot be calculated from available statistics, but interquartile ranges or ranges are reported, we will use the formula in Wan et al. [64] to estimate approximate standard deviations. When neither of the above methods are possible, we will impute the standard deviation using the average standard deviation across trials included in the same meta-analysis that have used the same measurement tool. When means are missing, but medians are reported, we will use the formula in Wan et al. [64] to estimate approximate means.

Our approach for dealing with missing outcome data within the primary trials will be through sensitivity analyses, where trials judged to be at a high or unclear risk of bias will be excluded (see the ‘Data synthesis’ section). Risk of bias ‘due to missing outcome data’ is considered within the overall bias judgement for a trial.

#### Assessment of heterogeneity

We will assess statistical heterogeneity of the intervention effects visually by inspecting the overlap of confidence intervals on the forest plots, formally test for heterogeneity using the χ^2^ test (using a significance level of *α* = 0.1), and quantify heterogeneity using the *I*^2^ statistic [65].

#### Assessment of biases due to missing results

We will use a framework for assessing risk of bias due to missing results in which an assessment is made for each meta-analysis regarding the risk and potential impact of missing results from studies (termed ‘known-unknowns’) and the risk of missing studies (termed ‘unknown-unknowns’) [66]. We will use this framework to guide our assessments of whether there is ‘undetected’ or ‘suspected’ reporting bias for each of the comparisons in our GRADE assessment (see the ‘Summary of findings tables and assessment of the certainty of the body of evidence’ section).

In assessing ‘known-unknowns’, we will determine what trials meeting the inclusion criteria for the particular meta-analysis have missing results through examination of the publication’s methods section, trial registry entry (if available) and trial protocol (if available). We will make an assessment as to whether the missing result was potentially due the result itself (e.g. ‘not statistically significant’), and whether inclusion of the result could lead to a notable change in the meta-analysis (e.g. if the missing result is from a large trial). We will also assess the impact of missing results from studies reported in languages other than English that were judged as being likely to meet the eligibility criteria for each synthesis (see the ‘Types of studies’ and ‘Selection of studies’ sections).

In assessing ‘unknown-unknowns’, we will judge whether the trials not identified were likely to have results eligible for inclusion (e.g. for broad outcome domains such as ‘pain’, it is likely that for particular conditions, missing studies would have been eligible for inclusion). We will use funnel plots and contour-enhanced funnel plots to examine whether there is evidence of small-study effects [67]. If there is funnel plot asymmetry, we will undertake a sensitivity analysis to compare the combined effect estimated from the random-effects model (primary analysis) with that estimated from a fixed (common) effect model. If the random-effects estimate is importantly larger than the fixed-effect estimate, with no explanation for the difference (e.g. differences in clinical populations or intensity of the delivery of intervention between small and large trials, differences in risk of bias between small and large trials), then we will downgrade for ‘suspected’ reporting bias.

#### Data synthesis

##### Meta-analysis

Separate comparisons will be set up based on outcome domains agreed in the final framework (see Fig. 2 and Appendix 2 in Additional file 1 for indicative groupings). These comparisons will be stratified by the population groups in the final framework, the basis for which may relate to symptoms (e.g. chronic pain), treatment for an underlying condition (e.g. patients undergoing surgery) or the underlying condition (e.g. chronic insomnia, dementia) (see Fig. 2 and the ‘Types of participants’ section for indicative groupings). This approach to structuring the meta-analysis will yield an overall estimate of the effect of aromatherapy for the outcome (review objectives 1, 2 and 4), as well as estimates within each population group (review objective 3). Subgroup analysis by population group will allow examination of whether these population groups explain any observed statistical heterogeneity in the intervention effects (see the ‘Subgroup analysis and investigation of heterogeneity’ section).

We will combine the effects using a random-effects meta-analysis model, since we expect there to be clinical and methodological diversity across the trials that may lead to statistical heterogeneity. These analyses will use the restricted maximum likelihood estimator (REML) of between trial heterogeneity variance and the Hartung-Knapp-Sidik-Jonkman confidence interval method.

Forest plots will be used to visually depict the intervention effect estimates and their confidence intervals. Forest plots will be stratified by condition and risk of bias (within population group).

##### Summary and synthesis when meta-analysis is not possible

Available effect estimates (95% confidence intervals, *p*-values), details of scales (direction and range), risk of bias assessments and intervention characteristics will be tabulated. Tables will be ordered by outcome domain, population group and risk of bias assessment.

For a particular comparison, if we are unable to analyse most of the effect estimates (due to incomplete reporting of effects and their variances, variability in the effect measures across the studies), we will consider alternative synthesis methods, such as calculating summary statistics of the effect estimates, combining *p*-values or vote counting based on the direction of effect [68]. Our choice of method will be determined by the available data (e.g. summary statistics if data permit; other methods if the data are more limited).

##### Subgroup analysis and investigation of heterogeneity

We will undertake a subgroup analysis to examine whether population group explains any observed statistical heterogeneity in the intervention effects (see Fig. 2 and the ‘Types of participants’ section for indicative population groupings and Appendix 2 in Additional file 1 for the subset of outcomes for which different population subgroups may be relevant). In addition, for the comparison aromatherapy versus inactive comparator, we will consider whether mode of delivery (massage or ‘other’) explains any observed statistical heterogeneity in the intervention effects.

##### Sensitivity analyses

We plan to undertake and report sensitivity analyses examining if the meta-analysis estimates are robust to the:

- *Meta-analysis model*. In addition to fitting a random-effects model, we will fit fixed-effect models. This analysis will be undertaken to investigate the impact of any small-study effects.
- *Inclusion of trials judged to be at an overall high or unclear risk of bias.* We will exclude trials judged to be at an overall high or unclear risk of bias.

Results of the sensitivity analyses will be tabulated, including the meta-analysis estimate (and its confidence interval), along with details of the original and sensitivity analysis assumptions.

#### Summary of findings tables and assessment of the certainty of the body of evidence

We will prepare GRADE summary of findings tables for each of the main comparisons, reporting results for critical and important outcome domains (up to seven). For each result, one author (SB) will use the GRADE approach to assess our confidence in where the effect lies relative to our threshold for a small effect (the certainty of evidence) (see the ‘Measures of treatment effect’ section). In accordance with detailed GRADE guidance [37, 69, 70], an overall GRADE of high, moderate, low or very low certainty will be reported for each result based on whether there are serious, very serious or no concerns in relation to each of the following domains.

1. Risk of bias. We will assess the overall risk of bias across all studies contributing to each synthesised result, considering the weight studies rated at high risk of bias contribute to the analysis. Serious or very serious concerns are more likely if studies at high risk of bias contribute considerable weight in the analysis and sensitivity analyses indicate that removing these studies changes the size of the effect (see the ‘Sensitivity analyses’ section).
2. Inconsistency. We will assess whether there is important, unexplained inconsistency in results across studies considering the overlap of confidence intervals (non-overlap indicating potentially important differences in direction or size of effect), statistical measures that quantify and test for heterogeneity (I^2^ statistic, *χ*^2^ test) and, where relevant, results of subgroup analyses (see the ‘Assessment of heterogeneity’ section). Where a result is based on a single study, inconsistency will not be rated.
3. Imprecision. We will assess whether the confidence interval for each pooled effect estimate is wide (e.g. including a small effect and little or no difference, which would lead to different interpretations) and, for large effects, whether the sample size meets the optimal information size (based on number of events for binary outcomes; sample size for continuous outcomes). In judging imprecision, we will use our threshold specified for a small effect (see the ‘Measures of treatment effect’ section).
4. Indirectness. We will assess whether there are important differences between the characteristics of studies included in each synthesis and the question we are seeking to address, such that the effects observed may not apply to our question (i.e. the applicability of the evidence). For example, differences between the interventions delivered and aromatherapy practice in Australia that are likely to influence the size of effect.
5. Publication bias. Our judgement of suspected publication bias will be based on assessment of bias due to missing results (see the ‘Assessment of biases due to missing results’ section). In these assessments, we will consider the potential impact on each synthesised result of excluding studies in languages other than English.
6. Upgrading domains (large effect size, dose response gradient, opposing plausible residual confounding). There is no precedent for rating up the evidence from randomised trials; however, in principle, these domains apply to any body of evidence so are included here for completeness.

Using GRADE decision rules, we will derive an overall GRADE for the certainty of evidence for each result included in the summary of findings table [70]. A result from a body of evidence comprised of randomised trials begins as ‘high’ certainty evidence (score = 4) and can be rated down (−1 or −2) for serious or very concerns on any GRADE domain that reduces confidence that aromatherapy has at least a small effect (as determined by the pre-specified thresholds) [61, 69, 70].

Summary of findings tables will be prepared using the GRADEpro GDT software [71]. The tables will include:

- Estimates of the effects of aromatherapy reported as standardised mean differences, and for binary outcomes relative and absolute effects
- The overall GRADE (rating of certainty) and an explanation of the reason(s) for rating down (or up) [68]
- The study design(s), number of studies and number of participants contributing data
- A plain language statement interpreting the evidence for each comparison and outcome, following GRADE guidance for writing informative statements [72].

We will present the four levels of certainty of evidence in summary of findings tables with the following symbols and interpretations.

- High (⊕⊕⊕⊕): further research is very unlikely to change the confidence in the estimate of effect
- Moderate (⊕⊕⊕⊝): further research is likely to have an important impact in the confidence in the estimate of effect
- Low (⊕⊕⊝⊝): further research is very likely to have an important impact on our confidence in the estimate of effect and is likely to change the estimate
- Very low (⊕⊝⊝⊝): any estimate of effect is very uncertain

## Supplementary Information

**Additional file 1: Appendix 1.** Database search strategies. **Appendix 2.** Example outcome domain. **Appendix 3.** TIDieR domains and example of application in aromatherapy systematic review.

## Data Availability

This is a protocol for a systematic review and does not contain any data. Requests for other material should be sent to the corresponding author.

## Abbreviations

AMED: Allied and Complementary Medicine Database
CENTRAL: Cochrane Central Register of Controlled Trials
CINAHL: Cumulative Index of Nursing and Allied Health Literature
CM: Complementary medicine
COMET: Core Outcome Measures in Effectiveness Trials
DEFF: Design effect
GRADE: Grading of Recommendations, Assessment, Development and Evaluation
IAAMA: International Aromatherapy and Aromatic Medicine Association
ICC: Intra-cluster correlation
ICD-11: International Classification of Diseases 11th Revision
ICTRP: International Clinical Trials Registry Platform
MeSH: Medical Subject Headings
MID: Minimal important difference
NHMRC: National Health and Medical Research Council
NRSI: Non-randomised study of interventions
NTREAP: Natural Therapies Review Expert Advisory Panel
NTWC: Natural Therapies Working Committee
PICO: Population, intervention, comparator, outcome
PRACI: Practitioner Research and Collaboration Initiative
PRISMA: Preferred Reporting Items for Systematic review and Meta-Analyses
PRISMA-P: Preferred Reporting Items for Systematic review and Meta-Analyses Protocols
RCT: Randomised controlled trial
REML: Restricted maximum likelihood estimator
RR: Risk ratios
SMD: Standardised mean difference
TIDieR: Template for Intervention Description and Replication
TGA: Therapeutic Goods Administration
