# Supplementary material for: Effectiveness of aromatherapy for prevention or treatment of disease, medical or preclinical conditions, and injury: protocol for a systematic review and meta-analysis
Source: Syst Rev. 2022 Jul 26;11:148. doi: 10.1186/s13643-022-02015-1 (PMC9317467; doi:10.1186/s13643-022-02015-1)
Supplement: Supplementary file 1 — Additional file 1: Appendix 1. Database search strategies. Appendix 2. Example outcome domain. Appendix 3. TIDieR domains and example of application in aromatherapy systematic review. [file 13643_2022_2015_MOESM1_ESM.pdf]

Appendices: Effectiveness of aromatherapy for prevention or treatment of disease, medical or preclinical conditions, and injury: a systematic review protocol

Appendix 1: Database search strategies

Cochrane Central Register of Controlled Trials

#	Search strategy
1	MeSH descriptor: [Aromatherapy] explode all trees
2	MeSH descriptor: [Oils, Volatile] explode all trees
3	(aromather* or aroma or aromatic or ((essential or inhal* or diffus* or massag* or bergamot or cedar or chamomile or camomile or eucalyptus or frankincense or geranium or ginger or lavender or lemon or mandarin or marjoram or orange or peppermint or rose or rosemary or “tea tree” or “ti tree” or melaleuca or valerian) near (oil* or scent*))):ti,ab,kw (Word variations have been searched)
4	#1 or #2 or #3

PubMed

(Aromatherapy[Mesh] OR Oils, Volatile[Mesh] OR aromather* OR aroma OR aromatic OR ((essential OR inhal* OR diffus* OR massag* OR bergamot OR cedar OR chamomile OR camomile OR eucalyptus OR frankincense OR geranium OR ginger OR lavender OR lemon OR mandarin OR marjoram OR orange OR peppermint OR rose OR rosemary OR “tea tree” or “ti tree” OR melaleuca OR valerian) AND (oil OR oils OR scent*))) AND ((Clinical Trial[PT] OR trial[TI] OR randomi* OR randomly OR placebo) NOT systematic[SB]) AND 09/01/2020:3000[EDAT]

Limited to Pubmed-not-MEDLINE subset, all years

((aromather* OR aroma OR aromatic OR ((essential OR inhal* OR diffus* OR massag* OR bergamot OR cedar OR chamomile OR camomile OR eucalyptus OR frankincense OR geranium OR ginger OR lavender OR lemon OR mandarin OR marjoram OR orange OR peppermint OR rose OR rosemary OR “tea tree” or “ti tree” OR melaleuca OR valerian) AND (oil OR oils OR scent*))) AND ((trial[TI] OR randomi* OR randomly OR placebo) NOT systematic[SB])) AND pubmednotmedline[SB]

AMED via Ovid

#	Search strategy
1	exp Aroma therapy/
2	Oils volatile/
3	(aromather$ or aroma or aromatic or ((essential or inhal$ or diffus$ or massag$ or bergamot or cedar or chamomile or camomile or eucalyptus or frankincense or geranium or ginger or lavender or lemon or mandarin or marjoram or orange or peppermint or rose or rosemary or “tea tree” or “ti tree” or melaleuca or valerian) adj6 (oil$ or scent$))).af.

4	exp Clinical trials/
5	(trial or random$ or placebo).af.
6	(1 or 2 or 3) and (4 or 5)

Emcare via Ovid

#	Search strategy
1	exp Aromatherapy/
2	(aromather$ or aroma or aromatic or ((essential or inhal$ or diffus$ or massag$ or bergamot or cedar or chamomile or camomile or eucalyptus or frankincense or geranium or ginger or lavender or lemon or mandarin or marjoram or orange or peppermint or rose or rosemary or "tea tree" or "ti tree" or melaleuca or valerian) adj6 (oil$ or scent$))).af.
3	exp Clinical Trial/
4	trial.ti. or (randomi$ or randomly or placebo).af.
5	(1 or 2) and (3 or 4)
6	(review or meta-analysis).ti.
7	5 not 6

Appendix 2: Example outcome domain

Outcome domain	Example outcomes	Populations to which this domain might apply
Symptoms and side-effects		
Pain (chronic or acute)	Overall and domain specific measures (e.g. pain on walking, early acute postoperative pain)	Chronic pain (musculoskeletal or other), cancer and advanced disease (not amenable to cure), headache/migraine, labour (pregnancy), acute postoperative or procedural pain, other acute pain.
Nausea and vomiting	Severity, duration	Cancer and advanced disease (not amenable to cure), postoperative, pregnancy (morning sickness)
Sleep disturbance	Nocturnal sleep time number of nocturnal awakenings	Cancer and advanced disease (not amenable to cure), dementia, postoperative
Fatigue	Severity, duration	Cancer and advanced disease (not amenable to cure)
Emotional functioning / wellbeing	Ability to cope, worry, psychological status, affect	Chronic pain (musculoskeletal or other), cancer and advanced disease (not amenable to cure), stress, anxiety or mood disorders (as clinically diagnosed conditions), dementia
Health-related quality of life (QoL)	Condition specific and general QoL measures	Chronic pain (musculoskeletal or other), cancer and advanced disease (not amenable to cure), stress, anxiety or mood disorders, chronic insomnia, dementia
Condition-specific outcomes		
Sleep disturbance	Nocturnal sleep time number of nocturnal awakenings	Chronic or acute insomnia (as primary diagnosis)
Behavioural and psychological symptoms of dementia (BPSD)	Global measures, specific BPSD domains especially agitation, aggression	Dementia
Clinical signs of skin conditions and wound healing	Time to wound healing; acne severity scores	Minor acute wounds, skin conditions such as acne vulgaris

Appendix 3 TIDieR domains and example of application in aromatherapy systematic review

Characteristic	Description	Standard headings (bolded) and codes illustrated with an example*
Brief name	Name or phrase	Ginger
Why	Rationale, theory or goal of essential elements	Relief of chemotherapy induced symptoms
What	Materials (physical or informational)	**ESSENTIAL OIL**<br>**Common name(s) of source material**: ginger (GIN-106)<br>**Scientific name**: not reported<br>**Origin**: Madagascar<br>**Active ingredients (as identified by study authors)**: 64.23% sesquiterpenes, 18.5% monoterpenes, and 3.28% aldehydes<br>**Mode of production**: steam distilled from fresh ginger<br>**Other parameters (as identified by study authors)**: The aroma of ginger is described as spicy, sweet, and warm.<br>**CARRIER OR DISPERSANT**: None reported<br>**CO-INTERVENTIONS (code all that apply):** written information leaflet, educational material, other [specify], none reported**
	Procedures, activities or processes including enabling/supporting activities	**ADDITIONAL PROCEDURES (code mode of administration under 'how')**: none reported
Who provided	Intervention provider by category and their expertise, background and training	**Administered by a provider**: [Y/N] **Self-administered**: [Y/N]<br>**Provider** (as identified by authors, code all that apply)<br>○ Aromatherapist<br>○ Naturopath<br>○ Massage therapists<br>○ Other natural therapist [specify]<br>○ Nurse (clinically qualified)<br>○ Allied Health [specify e.g. physiotherapist]<br>○ General practitioner/ physician [specify]<br>○ Research staff<br>○ Other [specify]<br>○ Not reported<br>**Highest level of training** (for health-care providers only): postgraduate, bachelor, diploma, certificate, other [specify], not reported<br>**Trained in aromatherapy?** [Y/N/not reported]
How	Modes of delivery (route of administration)	**CODE** (choose all that apply): inhalation – personal inhaler, inhalation – passive diffusion, topical – without massage, topical – with massage, other [specify]<br>**Verbatim extract**: [participants] will use the aromatherapy inhalers. The [participants] will remove the cover of the aromatherapy inhaler, place the aromatherapy inhaler under their nose and inhale three times with deep breathing (i.e., three sniffs). After use, the inhaler should be capped to minimize dispersal of the scent.
Where	Type of location where intervention occurred	n/a – self administered
When and how much	Number of times delivered, over what period of time,	**Number of time delivered:** n/a

	number of sessions, their schedule, and their duration, intensity or dose	**Over what time period:** Not specified. Three chemotherapy treatment cycles (i.e., Study Cycle 1, Study Cycle 2, and Study Cycle 3).<br>**Number of sessions and schedule:** During each Study Cycle, [participants] start the aromatherapy inhaler on the day before the start of their chemotherapy treatment cycle (Day 0) and continue using the inhaler for the next six consecutive days (Day 1-Day 6), including the day he/she starts their chemotherapy treatment cycle.<br>**Duration of sessions:** n/a<br>**Intensity and dose** (including % dilution if applicable)**:** 3 sniffs of the aromatherapy inhaler four times daily (morning, noon, evening, bedtime).
Tailoring	If personalised, titrated or adapted describe what, why, when and how	n/a
Modifications	Not collected. Any modifications are likely to be part of protocol (i.e. tailoring)	
How well	Not collected. Used for questions about *adherence* to intervention (not assignment to intervention)	

* Example from https://clinicaltrials.gov/ct2/show/NCT02670941

** Codes highlighted in grey are the codes that apply to this example
